# A comparative study of workability classes using seven case studies of engineering-geological investigations of sewer systems in Northern Moravia

**DOI:** 10.1038/s41598-023-40461-6

**Published:** 2023-08-16

**Authors:** Marian Marschalko, Zofia Zięba, Jan Kubáč, Kateřina Růžičková, Simona Matušková, Jolanta Dąbrowska, David Sysala

**Affiliations:** 1 Department of Geological Engineering, Faculty of Mining and Geology, VŠB-Technical University of Ostrava, 708 33 Ostrava, Czech Republic; 2 Department of Civil Engineering, Faculty of Environmental Engineering and Geodesy, Wrocław University of Environmental and Life Sciences, 50-365 Wrocław, Poland; 3 Department of Geoinformatics, Faculty of Mining and Geology, VŠB-Technical University of Ostrava, 708 33 Ostrava, Czech Republic; 4 Department of Environmental Engineering, Faculty of Mining and Geology, VŠB-Technical University of Ostrava, 708 33 Ostrava, Czech Republic

**Keywords:** Materials science, Civil engineering

## Abstract

While the main focus of numerous engineering-geological investigations is to determine load-bearing capacity and settlement in engineering structures, this article aims to point at the specificity of sewer system engineering-geological investigations, which focuses on workability of soils and rocks. The study deals with workability class assessment of seven different sewer system localities. The significance of this research lies in the mutual comparison of workability class assessment of these seven localities in terms of two different workability standards. Each of the standards represents an independent model of assessment and classification of workability. The first standard (CSN 73 1001) classifies soils and rocks into seven workability classes, while the second (EN ISO 14688) comprises only three workability classes. Each of the approaches has its advantages and disadvantages. In comparison to the first one, the second standard permits faster and easier classification of rocks, but may be less fair to investors or developers when considering the real engineering-geological conditions and costs of implementation. Rocks were newly classified into three (easy, medium and difficult) categories of earthwork realization difficulty. In the study, 33 layers were classified in the category of easy realization of earthworks, 8 layers in the category of a medium degree of earthwork realization difficulty, and two layers in the category of a difficult realization of earthworks.

## Introduction

One of the most interesting specificities of engineering geology is the fact that specific engineering-geological investigations have different goals, and thus use various methodologies, face unique problems, and have varying results in implementation. The basic boundary condition is the difference in the physical action of various structures (interaction) on specific engineering-geological conditions (subsoil) of the area.

Workability is a property which most resembles the breaking characteristic of rocks. It was discussed by Marschalko et al.^1–3^, Machniak and Kozioł^4^, Dunčková et al.^5^, and the breaking characteristic of rocks was dealt with by Commend et al.^6^, Golshani et al.^7^, Zhang et al.^8^, Zou^9^, Gong et al.^10^, and Cheng et al.^11,12^. Their research shows that the use of workability or breaking characteristic of rocks is given by the habitual practice in the construction industry and standards in the different countries. In contrast to the breaking characteristic of rocks, workability also includes excavated rocks loading onto a vehicle. This means that workability, similarly to the breaking characteristic of rocks, depends on the rock’s resistance to its loosening. Moreover, it also depends on the stickiness of rocks to the tools, soil loosening, or rocks’ resistance during loading or unloading during transport.

This article concentrates on the specificity in the assessment of sewer system engineering-geological investigations^13,14^. When compared to other engineering structures, in which investigations mostly aim to determine engineering-geological characteristics of load-bearing capacity and settlement, the focus in sewer systems is elsewhere. This is because the physical influence of sewer systems is completely different to other structures, and there is no need to deal with the load of the structure on the subsoil. In sewer systems, the difference in focus^15–18^ lies in the assessment of engineering-geological conditions of a locality in terms of workability, which is related to the feasibility of earthwork (excavations and backfilling)^19–22^. Earthwork represents the largest volume of all work when constructing sewer systems, and the goal of a construction project is to place an engineering structure (sewer system) into the subsoil. This means that workability is the most important boundary condition. It is also noteworthy that such construction works are the most frequent. Sewer systems are being restructured all across Europe and in many other parts of the globe as the environmental conditions need to be improved^23–25^. Therefore, it is important to better understand workability and the implementation of sewer system construction.

## Results and discussion

### Location and geological conditions of the case studies

**Figure 1:**
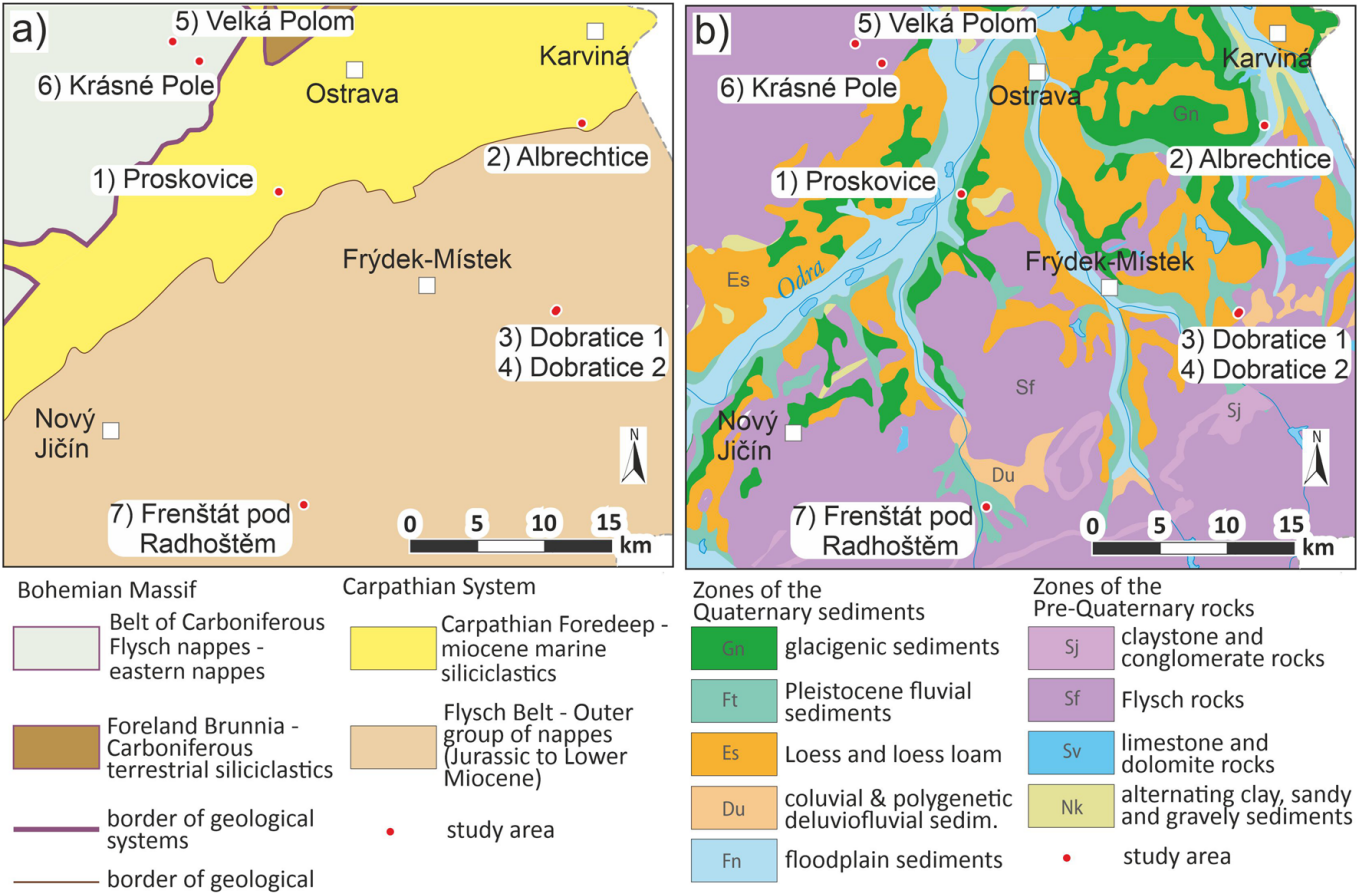
Location of the 7 case studies in the north-east of Czechia; (**a**) map of geological classification, (**b**) map of engineering-geological zones.

The research was implemented via seven case studies***,*** whose location is shown in the map of geological classification (Fig. 1a) and the map of engineering-geological zones (Fig. 1b) in Northern Moravia (north-east of Czechia). Locality 1 (Proskovice) is situated in the Carpathian Foredeep Miocene marine clay sediments in terms of the deeper geological structure. As for the surface geological structure of engineering-geological zones, the locality is found in the zone of Pleistocene fluvial sediments. Considering the deeper geological structure, locality 2 (Frenštát pod Radhoštěm) finds itself in the Cretaceous marine clayey and sandy sediments of Hradiště Formation. As for the surface geological structure of engineering-geological zones, the locality is found in the zone of Flysch rocks. Locality 3 and locality 4 (Dobratice 1, Dobratice 2) are situated in Upper Cretaceous marine clayey and sandy sediments of the Flysch Belt (Němčice and Frýdlant Formation) in terms of the deeper geological structure. As for the surface geological structure of engineering-geological zones, both localities are found in the zone of Pleistocene fluvial sediments. Locality 5 and locality 6 (Velká Polom, Krásné Pole) are situated in the Belt of Carboniferous Flysch marine clayey and wacke sediments of the Hradec-Kyjovice Formation. As for the surface geological structure of engineering-geological zones, both localities are found in the zone of Flysch rocks. Locality 7 Albrechtice is located in the Carpathian Foredeep Miocene marine clayey sediments in terms of the deeper geological structure. As for the surface geological structure of engineering-geological zones, the locality is found in the zone of Pleistocene fluvial sediments and floodplain sediments.

### Description of basic problems of engineering-geological investigations in the case studies

**Figure 2:**
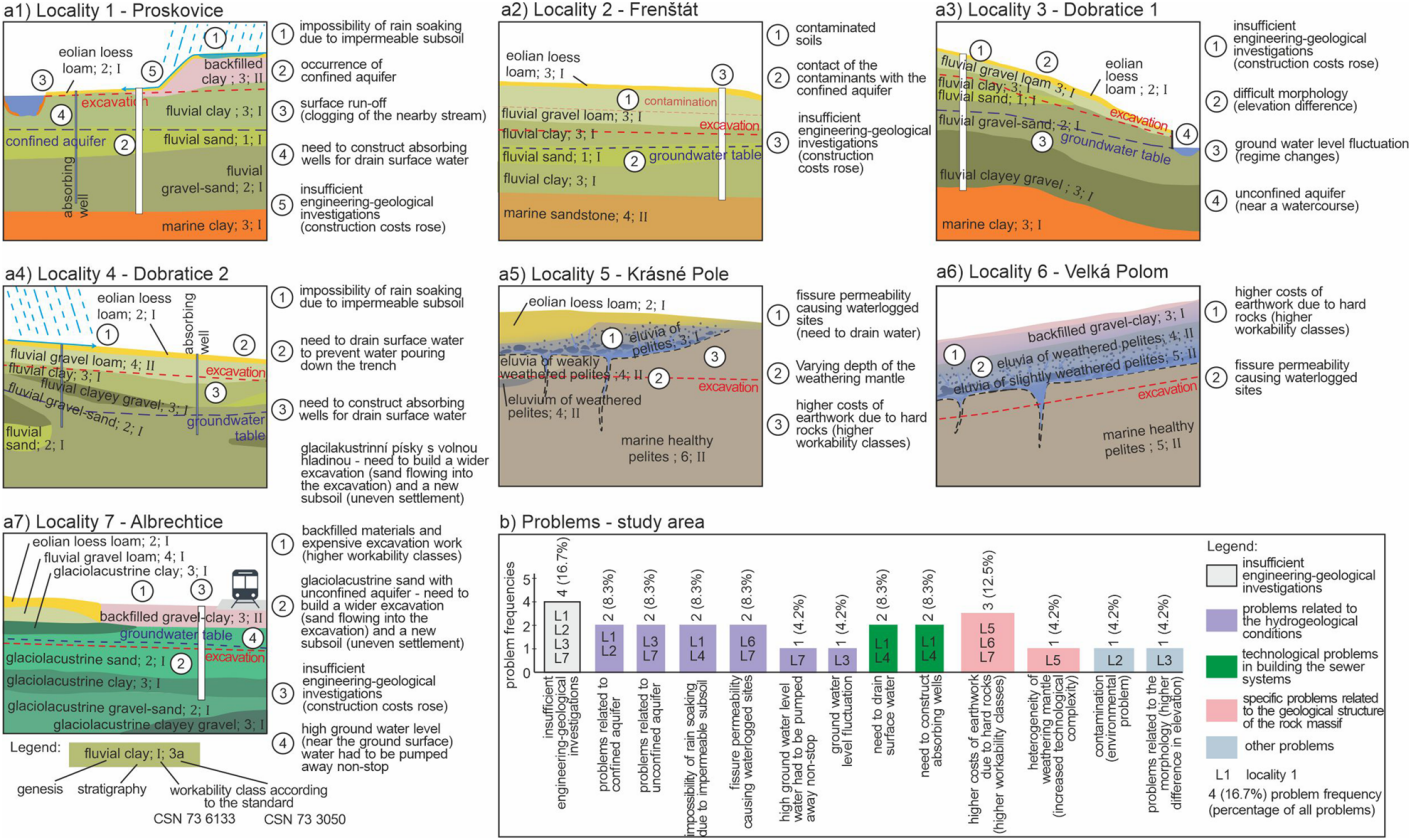
Description of basic problems in engineering-geological investigations and construction of the sewer systems depicted in engineering-geological sections; (**a1**) Proskovice, (**a2**) Frenštát pod Radhoštěm, (**a3**) Dobratice 1, (**a4**) Dobratice 2, (**a5**) Krásné Pole, (**a6**) Velká Polom, (**a7**) Albrechtice, (**b**) graph of problem frequencies in the localities.

In the case studies, the different layers were designated in line with the first standard (CSN 73 3050^26^), where the seven workability classes are marked with Arabic numerals (1, 2, 3, 4, 5, 6, 7). At the same time, each layer is classified into three workability classes in line with the second standard (CSN 73 6133^27^), where the workability classes are marked with Roman numerals (I, II, III). This distinction is used to point at the differences in workability classifications based on the two standards. The following paragraphs describe the case studies (see the engineering-geological section in Fig. 2) as for the engineering-geological structure with marked workability classes in line with the first and second standard and marked engineering-geological problems (Fig. 2) which occurred in the case studies.

The first case study in Proskovice (Fig. 2a1) was characterized by an engineering-geological section, where the first excavated layer in the direction from the ground surface is anthropogenic backfill of workability class 3 in line with standard 1 (CSN 73 3050^26^) and workability class I according to standard 2 (CSN 73 6133^27^). The second partially excavated layer is made up by Quaternary fluvial clay of workability class 3 (standard 1) and workability class I (standard 2). Although the other subsoil layers were not excavated, their characteristics were as follows: the third layer was fluvial sand (workability class 1, and I), the fourth was fluvial gravel-sand (workability class 2, and I), and the fifth layer was Neogene marine clay (workability class 3, and I). In this case study, the implementation of earthwork in connection with the sewer system construction project encountered a number of engineering-geological problems. The main problem was that the excavations were executed in the backfilled Neogene clay constituted by impermeable clay sediments, which meant that precipitation could not soak into the geological subsoil. This caused substantial surface run-off carrying clay material and resulted in the clogging of a nearby stream by sediments. Therefore, absorbing wells had to be constructed to drain surface water. Another engineering-geological complication was the occurrence of confined aquifer. Its base was formed by impermeable Neogene marine clays and the roof by impermeable fluvial clay. The lack of knowledge in the first phase of the engineering-geological investigations meant that the construction project incurred costs higher by 25%.

The second case study in Frenštát pod Radhoštěm (Fig. 2a2) was characterized by engineering-geological conditions, where the first excavated layer was eolian loess loam of workability class 3 (standard 1) and workability class I (standard 2). The second excavated layer was made up by fluvial gravel loam of workability class 3 and I. The third partially excavated layer was fluvial clay of workability class 3 and I. The other subsoil layers that were not excavated were fluvial sand (workability class 1 and I), fluvial clay (workability class 3 and I) and marine sandstone (workability class 4, and II). The engineering-geological problems in the second case study were specific because of the contaminated soils that were excavated during the construction project and had to be stored on a controlled landfill supervised by the Czech Environmental Inspectorate. It was crucial to prevent contact of the contaminants with the confined aquifer in the fluvial sediments. Because well-informed engineering-geological investigation had not been implemented before the project planning, the overall costs of the construction project rose by 30%.

The third case study in Dobratice (Fig. 2a3) was characterized by eolian loess loam of workability class 2 and I in the first excavated layer. The second excavated layer was made up by fluvial gravel loam of workability class 3 and I. The third partially excavated layer was constituted by fluvial clay of workability class 3 and I. The other layers (not excavated) were fluvial gravel-sand (workability class of 2 and I), fluvial clayey gravel (workability class 3 and I) and marine clay (workability class 3 and I). The engineering-geological conditions of the case study were specific because of the difficult morphology. During the excavation works, there was an elevation difference of 8 m on 100 m of the excavation. Because the slope dip was not parallel to the layer geometry, the excavation crossed three different geological environments. When the excavation reached fluvial gravel-sand, the natural environment had to be replaced with a 350-mm layer of aggregate (fractions 32/63 and 0/16 mm) because of the unconfined aquifer (near a watercourse), where the water level fluctuated by 1 m. The introduced aggregate layer was supposed to drain water and prevent uneven settlement. Another problem occurred with a wastewater treatment plant, to which the new sewer system was connected, and which had to be placed into saturated gravel-sand environment. A sealing reinforced concrete tank had to be constructed there. Similarly to the case studies above, due to insufficient engineering-geological investigations, the construction costs rose by 20%.

The fourth case study (Fig. 2a4) is also situated in Dobratice, but falls into a different cadastral district. The first excavated layer was made up by eolian loess loam of workability class 2 (standard 1), and workability class I (standard 2). The second excavated layer constituted of fluvial gravel loam of workability class 4 and I; the third partially excavated layer was fluvial clay of workability class 3 and I. Other subsoil layers, which were not excavated, were made up by fluvial clayey gravel (workability class 3 and I), fluvial gravel-sand (workability class 2 and I) and fluvial sand (workability class 2 and I). When implementing earthwork in connection with this sewer system construction, it was vital to deal with a number of problems arising from the engineering-geological conditions. There was an extensive surface run-off caused by impermeable eolian loess sediments. During heavier rain, surface run-off poured into trenches. At the same time, the developer had to modify drainage conditions along one part of the constructed sewer system. If we need to alter the hydrogeological conditions of a larger area, we should also deal with the conflicts possibly arising due to the existing wells and other engineering structures. It is always important to reduce the influence of the modified hydrogeological conditions in relation to the existing wells in the interest area. This means that the developer had to arrive at mutual agreements with the affected well owners. Next, absorbing wells with sufficiently permeable capacity had to be established. The investigations showed that fluvial gravel-sand would be most suitable. Another specificity was the depth of the wells (6–8 m) which had to be constructed under the supervision of the Czech Mining Institute. All the stated engineering-geological problems had to be considered and dealt with during earthwork.

The fifth case study in Krásné Pole (Fig. 2a5) was specific because of the geological structure of rocks, which manifested in the occurrence of higher workability classes. The first excavated layer constituted of eolian loess loam of workability class 2 and I; the second were eluvia of pelites of workability class 3 and I. The third excavated layer were eluvia of weakly weathered pelites of workability class 4 and II. The non-excavated layer, below the above stated layers, was an eluvium of weathered pelites of workability class 4 and II (this layer was divided into two quasi-homogeneous blocks). The last excavated layer contained marine healthy pelites of workability class 6 and II. In terms of hydrogeological conditions there was dominant fissure permeability bound onto rocks (marine pelites), which meant there were more saturated sites nearby the fissure system. The weathering mantle had a varying depth. As for the vertical heterogeneity, the upper layers contained rocks of lower workability classes than the deeper ones, because there were healthier rocks in the deeper parts (higher workability classes), and more weathered rocks higher up (lower workability classes). In terms of horizontal heterogeneity, the parameters may change in relation to the varying depth of the weathering mantle. Both the heterogeneities (horizontal and vertical) need to be considered when implementing earthwork as they represent increased technological intensity of earthwork.

The sixth case study in Velká Polom (Fig. 2a6) was characterized by similar geological structure as above. In the first excavated layer, there were backfilled materials of workability class 3 and I; in the second layer there were eluvia of weathered pelites of workability class 4 and II, followed by eluvia of slightly weathered pelites of workability class 5 and II, and a partially excavated layer of marine healthy pelites of workability class 6 and II. This meant that the engineering-geological investigation and earthwork implementation in connection with the sewer system construction were similar to the fifth case study. There was a rock massif, where the bottom part contained healthy and non-weathered rocks and the upper part contained a weathering mantle with semi-rocks and foundation loam soils. This meant that there were higher workability classes deeper down. This locality encountered similar problems as those mentioned above, which were related to the existence of fissure aquifers causing increased inflows of water into the construction site. However, the composition of the weathering mantle was even as for its thickness.

The seventh case study in Albrechtice (Fig. 2a7) had the following excavated layers: eolian loess loam (workability class 2 and I), backfilled gravel-clay (workability class 3 and I), fluvial gravel loam (workability class 4 and II), glaciolacustrine clay (workability class 3 and I) and partially excavated glaciolacustrine sand (workability class 2 and I). Among layers that were not excavated, there were glaciolacustrine clay (workability class 3 and I), glaciolacustrine gravel-sand (workability class 2 and I) and glaciolacustrine clayey gravel (workability class 3 and I). The specificity of this project were anthropogenic backfills in the upper layer that had a higher workability class than the lower layers. This fact made the earthwork more expensive than if there had been the original natural geological structure (no backfilled materials). Another complication was the implementation of excavation in the glaciolacustrine sand in combination with the existence of an unconfined aquifer on the same level (circa 2 m below the ground surface). At such sites, sand poured from the sides into a trench. Thus, the width of the excavation was 4 m instead of 1.5 m. Another complication in this case study was the existence of a railway track and the need to pipe-burst an underground collector with an insertion pit and reception pit. These operations had to be observed by the Czech Mining Institute. Another engineering-geological problem was the fact that the saturated glaciolacustrine sand represented unstable subsoil for sewer systems, with the risk of differential settlement. Thus, a 50-cm layer below the pipes had to be replaced with aggregates (fraction 32/63 and 0/16). The existence of an unconfined aquifer at the depth of 2 m meant that water had to be pumped away non-stop as earthwork went as deep as 3.5 m. It is worth noting that a usual progress when constructing sewer systems in better engineering-geological conditions is 15–20 m a day. However, in this case it was only 6 m, as trench boxes had to be used for earthwork. The initial engineering-geological investigations were underestimated which meant that the project costs were higher by 40%.

For clarity and aggregation of the basic problems encountered during engineering-geological investigations in the case studies, we plotted a graph with different engineering-geological problem frequencies (Fig. 2b) when constructing the sewer systems. The most important experience was that the initial engineering-geological investigations were underestimated and caused substantial increases in the construction costs. This concerned four out of the seven localities, and the increase ranged from 20 to 40%. Another group of problems was related to the hydrogeological conditions. The complications were unconfined aquifers in two of the localities (8.3%—expressed as a percentage of all problems at the seven case studies), confined aquifers in two localities (8.3%), impossibility of rain soaking due to impermeable subsoil in two localities (8.3%), ground water level fluctuation in one locality (4.2%), fissure permeability causing waterlogged sites in two localities (8.3%) and high ground water level in one locality (4.2%). Another group of problems were technological problems in building the sewer systems (frequency 4, percentage 16.7%). These were, for example, the need to construct absorbing wells in two localities (8.3%) and the need to drain surface water to prevent water pouring down the trench in two localities (8.3%). The next group of problems was related to the specific geological structure of the rock massif in four localities (16.7%). This meant the developers had to deal with the heterogeneity of the weathering mantle (heterogeneity of workability classes = higher technological demands) at one locality (4.2%), and higher costs of earthwork due to hard rocks (higher workability classes) in three localities (12.5%). Other problems concerned the environmental burden of soil contamination and its replacement with new layers in one locality (4.2%) and difficult morphology related to higher difference in elevation in one locality (4.2%).

### Character of soil types in the case studies

**Figure 3:**
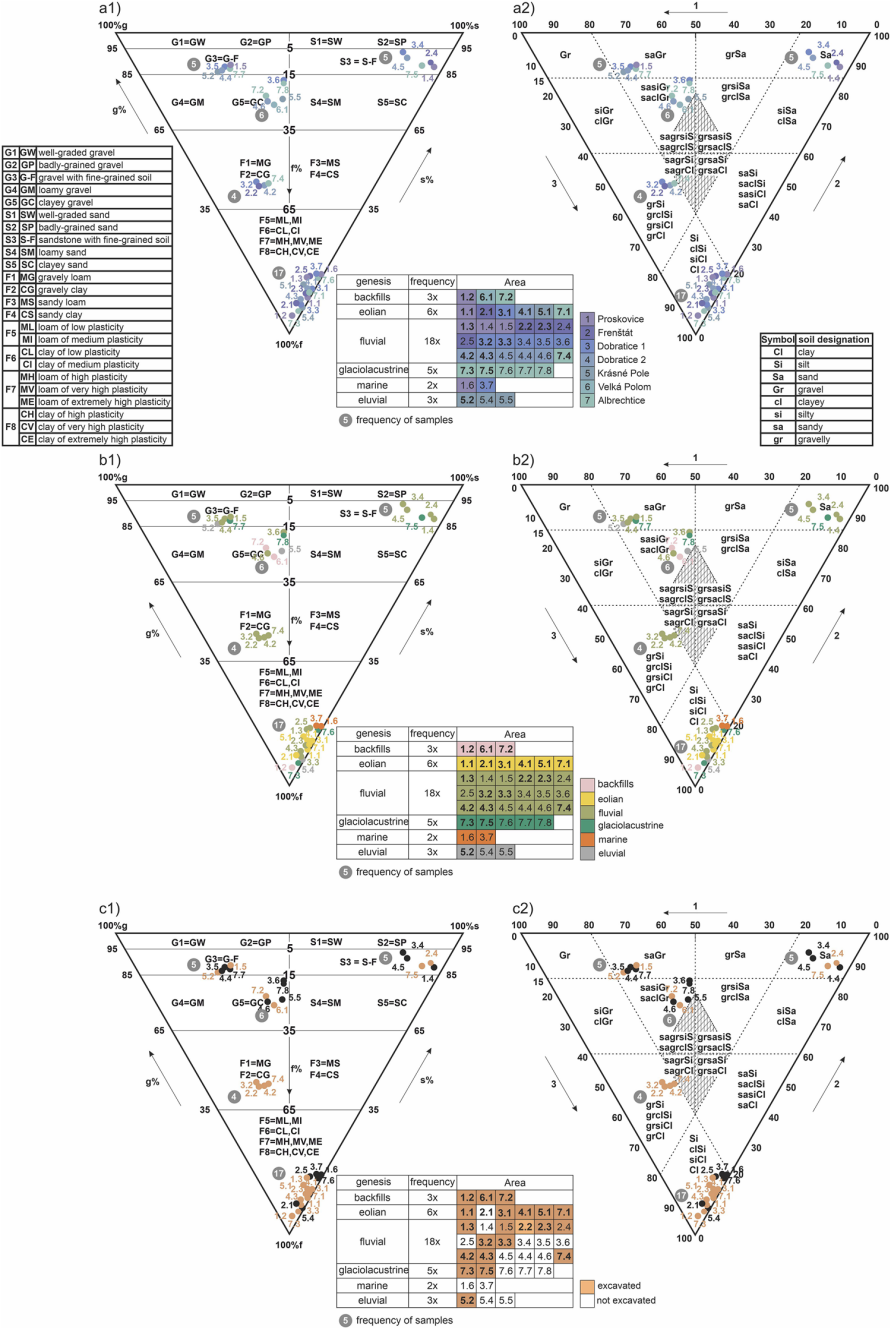
Soil types in the different layers of the seven localities (case studies); (**a**) according to the localities (case studies), (**b**) according to the genesis, (**c**) according to the criterion whether the layer was excavated or not; (**a1**,**b1**,**c1**) classification diagram according to the standard CSN 73 1001^28^, (**a2**,**b2**,**c3**) classification diagram according to the standard CSN EN ISO 14688-2 (72 1003)^29^.

If we want to systematically evaluate the workability classes, we need to identify the character of soils and rocks (soil and rock types) which will further be assessed for workability. Among the many reasons, one is that engineering-geological investigations help to classify foundation soils into soil types. Next, foundation soils are assessed for grain-size distribution. This is important for workability assessment described below. To make a classification system for all the localities (case studies) from the point of view of soil types, we sampled and classified each layer. The overall results from all the localities and laboratory tests are presented in Fig. 3. At the same time, all soil types were classified into two systems (triangles) considering the two standards, i.e., standard CSN 73 1001 (Foundation of structures—Subsoil under shallow foundations^28^)—Fig. 3a1,b1,c1 and standard CSN EN ISO 14688-2 (Geotechnical investigation and testing—Identification and classification of soil—Part 2: Principles for a classification^29^)—Fig. 3a2,b2,c2).

If we evaluate soil types according to the localities (case studies), there is not one dominant group of soil types typical for the localities (Fig. 3a1,a2). The most abundant are soil types F6(CL), F8(CH), G5(GC), G3(G-F), S3(S-F) and F1(MG). The most frequent are fine-grained soils with 21 layers, followed by gravel loams (11 layers) and the fewest are sandy loams (5).

When evaluating soil types as for genesis (Fig. 3b1,b2), it is clear that that there are 6 genetic types in the 7 localities. The most abundant group are fluvial sediments (18 layers). The other genetic types are glaciolacustrine, eolian, eluvial, anthropogenic and marine sediments. No genetic type of certain soil is dominant, except for the soil type F1(MG), i.e., one genetic type of fluvial sediments.

The last evaluated criterion (Fig. 3c1,c2) we also quantified in connection with the classification triangles of soil types was whether a soil type was excavated or not. Even if a layer was not excavated, it made part of the examined subsoil. We found that some genetic soil types were always excavated. For example, these were anthropogenic backfills that were found on the ground surface. On the contrary, marine sediments, as preQuaternary types, were hardly ever excavated as they are situated deeper. Usually, only Quaternary sediments were excavated.

### Classification system of workability according to the tools and mechanisms used in the case studies

**Figure 4:**
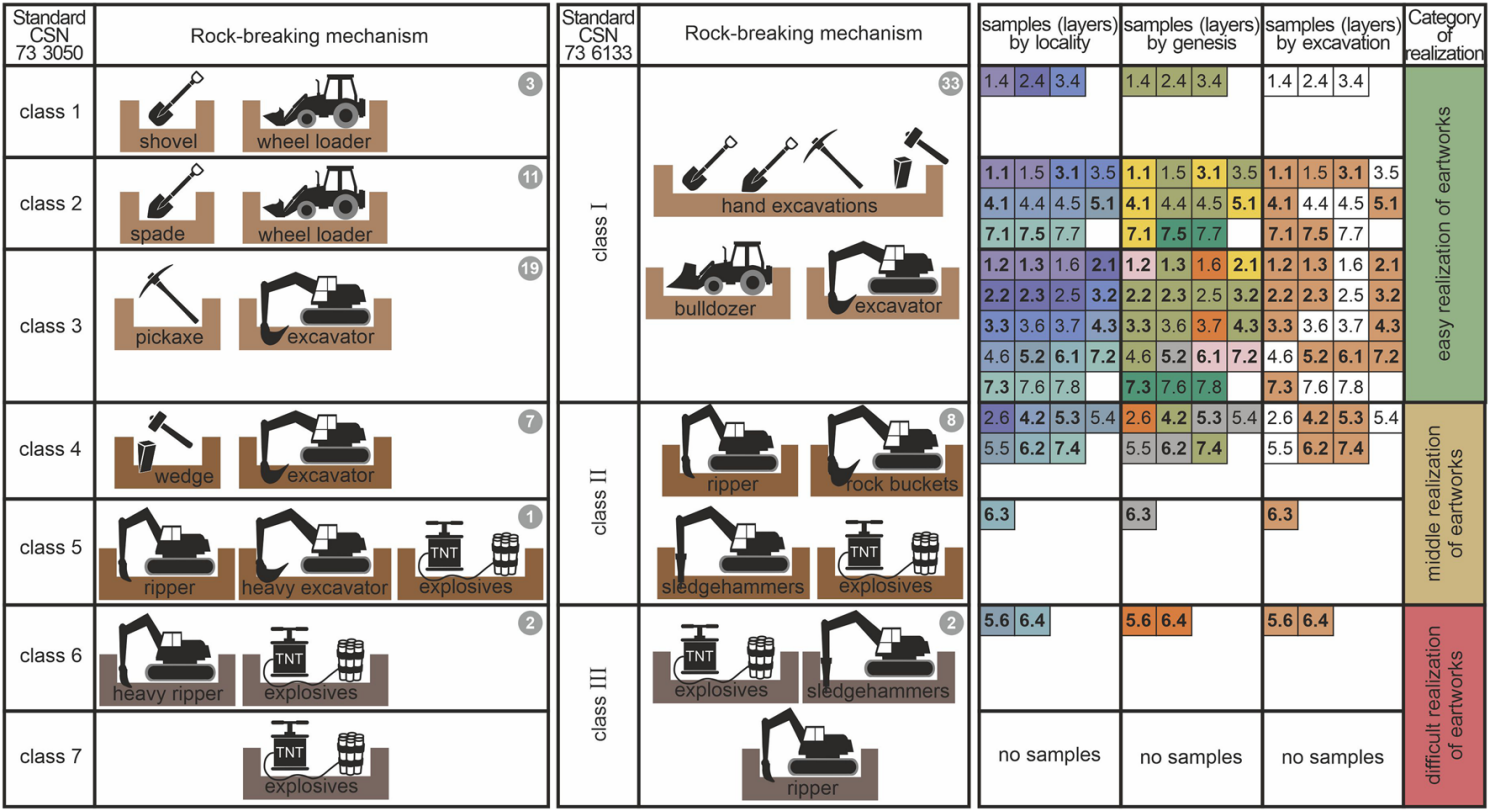
Comparative table of workability classes in line with the two standards and used tools and mechanisms during earthwork.

The most important factor as for workability is the relation of each workability class to the used tool or mechanism (Fig. 4). There are seven workability classes according to the first standard, and three according to the second standard, where each class is related to a characteristic tool or mechanism. The tools and mechanisms may fall into more classes because although the same mechanism is used for earthwork, different amount of work is spent on various workability classes (e.g. different plasticity, consistency of cohesionless soils; different relative compaction in cohesive soils; different planes of cleavage in hard rocks). This means that the costs of earthwork will also vary due to spent energy and tool wear.

According to the first standard, the first workability class (Fig. 4) is represented by cohesionless soils workable by a shovel (wheel loader). The second workability class is characterized by diggable soil that is workable by a spade (wheel loader). The third workability class is constituted by diggable soil workable by a pickaxe (excavator). If we look for an analogy with the second standard, the first workability class I includes classes 1, 2 and 3 under the first standard. The common characteristic of the groups is the fact that earthwork is carried out manually using tools or using common mechanisms, such as dozers and excavators. The fourth workability class (first standard) are friable soils workable by a wedge (excavator). The fifth workability class are solid rocks easy to blow, which are breakable by a ripper or heavy excavator (weight over 40 t) and explosives. The fourth and fifth class are analogous with the workability class under the second standard. The characteristic common feature is the fact that special breaking mechanisms, such as rippers, rock buckets and sledgehammers, are used for earthwork. The sixth workability class (the first standard) are solid rocks difficult to break by a heavy excavator. The seventh class are very hard rocks difficult to excavate, which are broken by explosives. The sixth and seventh class are analogous to workability class III under the second standard. For both groups it is typical that blasting must be used to break the rocks.

Based on the comparison of the both standards, it is possible to define new categories of earthwork realization degree of difficulty (three categories—easy, medium and difficult). The first category is the least costly due to simplest technologies applied. This easy earthwork realization degree is related to workability classes 1, 2 and 3 (according to the first standard—CSN 73 3050^26^) and workability class I (according to the second standard—CSN 73 6133^27^). The second category is the category of medium earthwork realization degree related to classes 4 and 5 (according to the first standard—CSN 73 3050^26^) and workability class II (according to the second standard—CSN 73 6133^27^). The last and most costly and most demanding category is reserved for workability classes 6 and 7 (according to the first standard—CSN 73 3050^26^) and class III (according to the second standard—CSN 73 6133^27^).

Figure 4 shows the frequency of the different layers (samples) in the case studies according to the location, genesis of the different layers, and whether the layers were excavated or not (made part of the engineering-geological investigations). The most layers fall into the workability class 3 (19 layers of different genetic types) and 33 layers in workability class I in line with the second standard. The second most frequent group (as for the first standard) was workability class 2 (11 layers), again made up by different genetic types. This means that the trend of alternating genetic types is similar in the most frequent workability class groups based on the tools and mechanisms.

### Classification system of workability classes according to the rock property criteria used in the case studies

**Figure 5:**
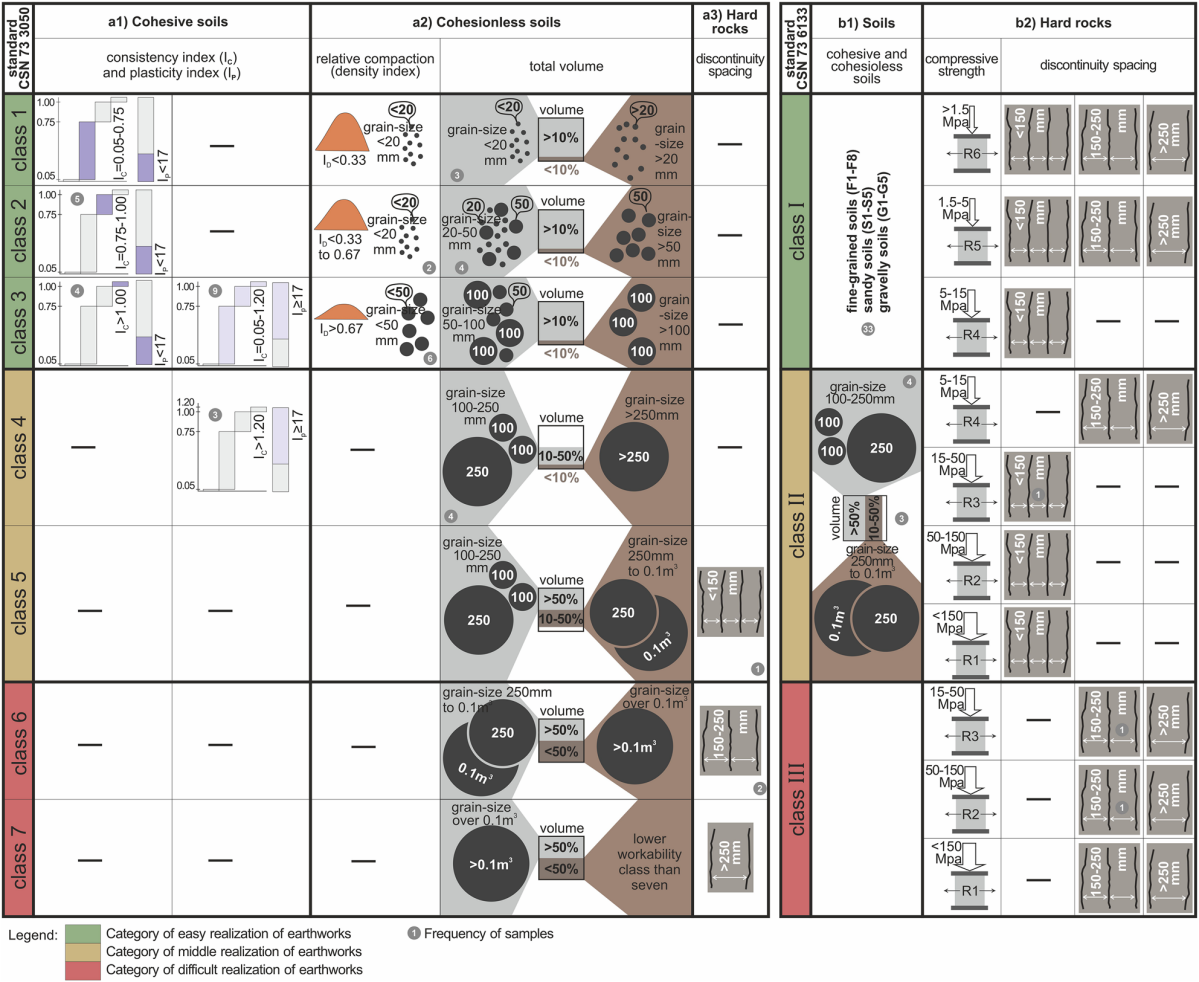
Graphic representation of workability classification based on classification criteria (soil and rock properties); (**a**) in line with standard 1—CSN 73 3050, (**a1**) cohesive soils, (**a2**) cohesionless soils, (**a3**) rocks; (**b**) in line with standard 2—CSN 73 6133, (**b1**) soils, (**b2**) hard rocks.

In order to better understand the classification system of workability classes considering the different criteria of rock properties applied in the case studies, we must first compare the 2 standards that classify the soil and rock workability classes (Fig. 5). Each standard approaches the workability classification differently although some criteria are analogous.

The first standard *CSN 73 3050 *^26^ classifies soils and rocks based on three types, i.e. cohesive soils, cohesionless soils, and hard rocks. In the case of cohesive soils (Fig. 5a1), the combined assessment criterion is the consistency index and plasticity index. An important characteristic to determine workability in cohesive soils is natural water content^30–32^, which enters the consistency index calculation. In the case of cohesionless soils (Fig. 5a2), the first assessment combined criterion is relative compaction (density index) and certain grain-size ratio. This combined criterion may be applied in workability classes 1–3 only. Next, there is the second combined criterion to assess cohesionless soils (Fig. 5a2), which is the combination of partial volume percentage (expressed in per cents; there are usually two volume percentages, the sum of which is 100%) out of the overall volume percentage of a particular grain-size fraction. This combined criterion may be applied in all workability classes 1–7. In the case of hard rocks (Fig. 5a3), the assessment criterion is the discontinuity spacing, which creates certain blocks allowing for loading during earthwork.

Cohesive soils (Fig. 5a1) of plasticity index below 17 fall in the first group. This group contains workability class 1 (at consistency index 0.05–0.75) and 2 (at consistency index 0.75–1.00). Workability class 3 is specific, as the plasticity index can be even below 17. Reaching plasticity index below 17 (part of first group), the consistency index must be over 1.00, and at plasticity index over 17 (part of the second group) the consistency index must be between 0.05 and 1.20. The remaining part of the second group is workability class 4, where the plasticity index is over 17 and the consistency index is over 1.20. As for the workability classes 5, 6 and 7, these cannot be used for cohesive soils.

Cohesionless soils (Fig. 5a2) are classified into workability classes based on two criteria. The first criterion is the relative compaction (density index), which is used to classify soils of workability classes 1–3, but certain boundary conditions for grain-size distribution apply there (Fig. 5a2). The second criterion is the combined criterion is grain-size ratio (certain grain fractions) and volume percentage*.* The first workability class is characterized by volume percentage of cohesionless soils over 10% and grain-size distribution below 20 mm. The remaining part is cohesionless soils with volume percentage below 10% and grain-size distribution over 20 mm. The second workability class is characterized by the majority volume percentage of cohesionless soils over 10% and grain-size 20–50 mm. The minority part is made up by volume percentage below 10% and grain-size over 50 mm. The third workability class is given by the majority volume percentage over 10% and grain-size 50–100 mm, while the remaining minority part is made up by volume percentage below 10% and grain-size over 100 mm. The fourth workability class is specified by volume percentage of 10–50% and grain-size 100–250 mm, and at the same time, volume percentage below 10% and grain-size from 250 mm to 0.1 m^3^ (576-mm spherical grain diameter). The fifth workability class is characterized by volume percentage of cohesionless soils over 50% and grain-size from 250 mm to 0.1 m^3^, and at the same time, volume percentage 10–50% and grain-size of 100–250 mm. The sixth workability class is given by the volume percentage over 50% and grain-size over 0.1 m^3^, and at the same time, volume percentage below 50% and grain-size from 250 mm to 0.1 m^3^. The seventh workability class is given by the majority volume percentage over 50% of cohesionless soils of a lower workability class than seven, and at the same time, the minority volume percentage below 50% grain-size below 0.1 m^3^.

Hard rocks (Fig. 5a3) are classified into workability classes 5, 6 and 7. The difference is that in workability class 5 the discontinuity spacing is up to 150 mm in the fissure system; in workability class 6 it is from 150 to 250 mm, and in workability class 7 the discontinuity spacing is over 250 mm.

The second standard CSN 73 6133^27^, used to assess workability classes in the seven case studies, is more recent. Although the concept is simpler as for considering the properties, the problem is that it has only three workability classes. Contrary to the first standard, the second one distinguishes only two soil types (hard rocks and soils—it does not distinguish cohesive and cohesionless soils) based on which workability classes are classified.

The assessment criterion in soils is that the whole group of fine-grained, sandy and gravelly soils fall in workability class I, and cohesionless soils of larger grains (stony and boulder soils) fall in the workability class II (Fig. 5b1).

As for the assessment of hard rocks (Fig. 5b2), the combined criterion is compressive strength (hard rock groups R1-R6 based on compressive strength) and discontinuity spacing. The weakest are groups R5 and R6 and they fall in workability class I regardless of the discontinuity spacing. However, there are two workability classes for group R4. Workability class I is for discontinuity spacing up to 150 mm, and workability class II for discontinuity spacing over 150 mm. As for the remaining groups (R3, R2 and R1), these may fall into workability class II or III. The difference is that in workability class II, the discontinuity spacing must be below 150 mm, and in workability class III discontinuity spacing must be over 150 mm.

There is a relationship between workability classes, consistency index, and plasticity index of cohesive soils. This relationship is important as a classification criterion to classify cohesive soils—see Figs. 5a1 and 6a which shows the discrete layers of case studies. The x-axis gives the workability classes and y-axis the consistency index. Each of the evaluated columns is supplied with a boundary condition of a plasticity index value. This way, we can see two groups of columns, where the first group is characterized by plasticity index below 17 (dark violet), and in the second group, the plasticity index is equal to or higher than 17 (light violet).

**Figure 6:**
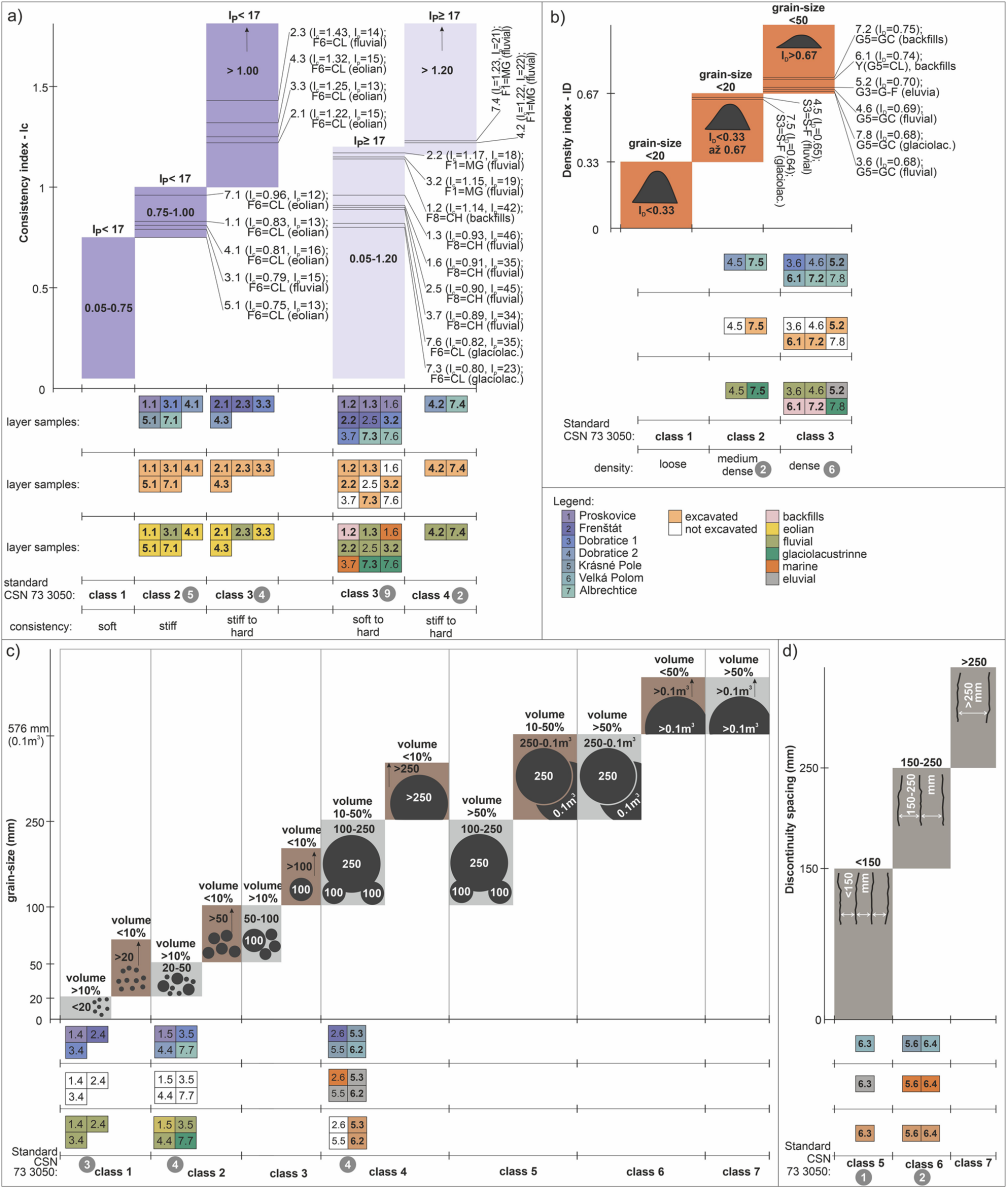
Evaluation of workability classes in the case study layers based on soil and rock properties (standard 1); (**a**) based on consistency index and plasticity index of cohesive soils, (**b**) based on combined criterion of relative compaction (density index) and grain-size distribution of cohesionless soils, (**c**) based on combined criterion of grain-size distribution and volume percentage of cohesionless soils, (**d**) based on discontinuity spacing in hard rocks.

The relationship between workability, consistency index, and index of plasticity is clear and logical. For example, in the case of fine-grained soil of soft consistency (Fig. 6a), it can be loaded using a shovel (Fig. 4), which is workability class 1. However, this applies if plasticity index is below 17 (and the consistency index is 0.05–0.75). If the plasticity index is equal to 17 (consistency index 0.05–1.20), the soft soil is workable by a pickaxe (Fig. 4), which is a workability class 3. In case of fine-grained soil of stiff to hard consistency (Fig. 6a) and plasticity index over 17 (consistency index over 1), the soil is workable by a pickaxe (workability class 3). On the contrary, if the plasticity index is over or equal to 17 (consistency index over 1.2), the soil is not workable by a pickaxe, and a wedge or sledgehammer must be used (workability class 4). However, when we use the second standard for evaluation, both the groups fall in workability class I.

This implies that the first standard is much more sensitive to the breaking characteristic of rocks (workability) of fine-grained soils. This means that the expected costs of earthwork (according to the first standard) corresponding to workability classes 1, 2, 3 or 4 may be more realistic in the sense of consistencies and index of plasticity. Changing the parameters, the intensity of machinery energy consumption and wear clearly differ. The second standard only states that all these soils fall in the same workability class I and can be dredged. This means that the estimations of costs according to the second standard will be more favourable for investors, but do not fully take into account the real conditions of the soil massif as for consistencies, plasticity index, or consistency index.

Out of the 43 assessed layers, the highest number were 9 layers (column 4 out of 5; Fig. 6a) in the group of plasticity index equal to or higher than 17 and consistency index of 0.05–1.20, which corresponds to soft to hard consistency and workability class 3 (first standard) and workability class I (second standard). The second highest frequency of 5 layers (column 2 out of 5; Fig. 6a) was in the group of consistency index 0.75–1.00 and plasticity index below, which corresponds to workability class 2 (first standard) and workability class I (second standard).

If we assess the different samples (layers) corresponding to the layers assessed by genesis, the most pronounced heterogeneity in properties was recorded in the fluvial type (Fig. 6a), which is found in every assessed group (the column with a particular index of consistency and plasticity). On the other hand, the least heterogeneous as for properties was the type of marine, glaciolacustrine and anthropogenic sediments, as it was found only in one group (consistency index 0.05–1.20; plasticity index ≥ 17). If we assess layers according to the factor whether they were excavated or not, the excavated layers occurred in all four groups, while the non-excavated layers (sewer system subsoil) were found in one group (Fig. 6a).

Among the workability classes and the combined criterion of relative compaction (density index) and certain grain-size ratio of cohesionless soils there is a relationship which is important as a workability classification criterion—see Fig. 5a2. There are three groups in the relative compaction index. For workability class 1, the relative compaction must be below 0.33, under the boundary condition that cohesionless soils grain-size distribution is below 20 mm. In workability class 2, the relative compaction is 0.33–0.67, under grain-size distribution below 20 mm. However, there are different boundary conditions for workability class 3. The relative compaction must be over 0.67 and grain-size distribution below 50 mm. The identified layers of cohesionless soils in the case studies are in Fig. 6b. Using these criteria, 8 layers were classified here. The most frequent were the 6 layers in workability class 3 and relative compaction over 0.67 (grains below 50 mm). The second were backfills two layers of workability class 2 and relative compaction 0.33–0.67 (grains below 20 mm).

Another classification criterion for cohesionless soils is the relationship of workability classes and combined criterion of grain-size and volume percentage. The general boundary conditions of this relationship are shown in Fig. 5a2 and stated above. The concrete results are in Fig. 6c. We found that the most frequent were two groups. The first group had four layers of workability class 2 (grain-size distribution of 20–50 mm in the volume percentage of over 10%, and grain-size distribution over 50 mm in the volume percentage below 10%). The second group had four layers of workability class 4 and was related to grain-size distribution of 100–250 mm in the volume percentage 10–50%, and grain-size distribution over 250 mm in the volume percentage below 10%. The remaining volume percentage was represented by soils of workability class 3. The last group of three layers had workability class 1 (grain-size distribution below 20 mm in the volume percentage over 10%, and grain-size distribution over 20 mm in the volume percentage below 10%).

Hard rocks (Fig. 6d) were classified into workability classes 5, 6 and 7 based on discontinuity spacing. The highest discontinuity spacing (over 250 mm) is characteristic of workability class 7. On the other hand, the lowest discontinuity spacing is below 150 mm, and only one such layer was identified in all the case studies. The workability class 6 is characterized by discontinuity spacing between 150 and 250 mm, and two layers fell in this class.

A separate graphical representation for the second standard was not produced because of a simple comparison in Fig. 5. It implies that the most frequent group of layers (33) had the character of fine-grained, sandy or gravelly soils (Fig. 5b1), and was thus classified into workability class I. The other layers were less frequent. An example are hard rocks, in which the combined criterion of compressive strength and discontinuity spacing was used.

### Quantification of degree of earthwork realization difficulty

**Figure 7:**
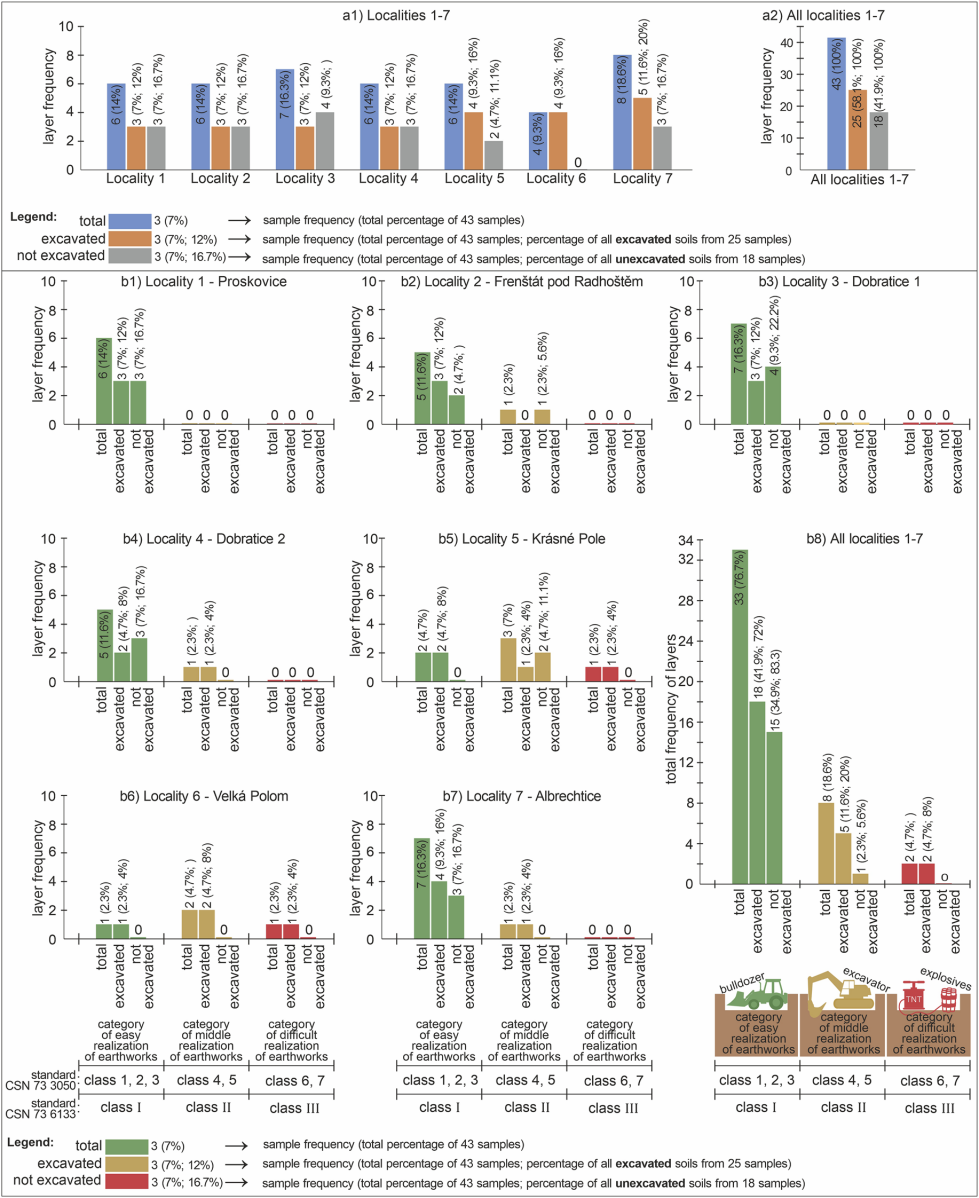
Identified frequencies of layers in the case studies; (**a**) layer frequency—(**a1**) in the discrete localities, (**a2**) in all the localities; (**b**) layer frequency based on the classification into new categories of degree of earthwork realization difficulty (easy, medium and difficult degree of earthwork realization)—(**b1**) Proskovice, (**b2**) Frenštát pod Radhoštěm, (**b3**) Dobratice 1, (**b4**) Dobratice 2, (**b5**) Krásné Pole, (**b6**) Velká Polom, (**b7**) Albrechtice, (**b8**) all localities.

The category of the degree of earthwork realization difficulty is defined in Figs. 4 and 5 and the text related to Fig. 4. The categories are as follows: an easy (green), medium (yellow) and difficult (red) degree of earthwork realization. The overall statistics of assessed layers (frequency of sampled layers) is presented in Fig. 7, while the quantification of the localities can be found in Fig. 7a1. It is also summarized in Fig. 7a2. These figures distinguish the different columns based on the overall number of the assessed layers (blue), the number of excavated layers (brown) and the layers that were investigated but were not excavated during the sewer system construction (grey, Fig. 7a). A similar system is used to show the layer frequency of earthwork realization, but the colours depend on the categories. At the same time, all the graphs were shown to display the case studies in Fig. 7b1–b8, and the overall state in Fig. 7b8.

The research shows that 76.7% of all layers (33 layers) are in the most favourable category (category of easy realization of earthwork) as for the cost and degree of difficulty of earthwork (Fig. 7b8). There were 41.9% (18 layers) that were excavated in this category. The share of excavated layers in this category on the overall number of excavated layers was 72%. This is logical because this category of recent, non-consolidated Quaternary geological structure, found near the ground surface, is used for sewer system construction (and other surface engineering structures). This geological structure corresponds to a lower degree of consolidation than those of preQuaternary rocks, which means that such rocks are easy to break and load (lower workability classes).

On the other hand, the least favourable category of difficult degree of earthwork realization (most costly and most technologically demanding) concerns 4.7% (two) layers out of all the assessed samples (Fig. 7b8). This is explained by the fact that only two localities are found in hard rock eluvia (localities 5 and 6). This negative (as for costs, energy intensity and technology demands) category is bound onto hard rocks, but their regional frequency is low. Another reason is the fact that hard rocks of R1 and R2 type (or exceptionally semi-rocks R3) rarely outcrop in Czechia, and thus do not largely concern the construction of sewer systems. This especially applies in Northern Moravia where in a large part of the area the preQuaternary structure is topped by thick Quaternary glaciolacustrine sediments.

In the category of medium degree of earthworks realization difficulty there are 18.6% (eight) layers out of all the assessed samples, while the excavated layers form only 11.6% (five) out of all the samples, and 20% out of all the excavated layers. It is most related to the genetic type of eluvial and marine sediments in the case studies.

If we assess the heterogeneity in the different categories of degree of earthwork realization difficulty in the studied localities, three facts can be established. The most homogeneous were two localities (locality 1—Proskovice, and locality 3—Dobratice 1), where only one category (easy realization of earthwork) was identified. There were only easily workable soils with corresponding engineering-geological properties. On the contrary, the most heterogeneous geological structure as for earthwork realization was in locality 5—Krásné Pole, and locality 6—Velká Polom, as all the categories were recorded there. This is because hard rocks are deeper in the weathering mantle. In the remaining localities (locality 2—Frenštát pod Radhoštěm, locality 4—Dobratice 2, and locality 7—Albrechtice) there were only two categories of earthwork realization difficulty, namely the category of easy and medium earthwork realization difficulty as no hard rocks were found.

## Conclusions

Soil and rock workability is one of the most important properties in engineering geology. When surface engineering structures are built, during which earthwork needs to be implemented, the costs of the construction and the technology intensity are at stake. This means that soil and rock workability is one of basic boundary conditions of each engineering-geological investigation. In sewer systems, it is a particularly important property as earthwork represents the most work. The research results show that the basics of each sewer system construction project should be a good engineering-geological survey, sufficient in its extent and quality. Otherwise, the costs may rise by 20–40% as was shown in this study.

When comparing the case studies, we find that five out of seven localities have loam type of soils. As for workability, the geological structure falls in workability classes 1 to 4 (standard 1) and class I (or II) in line with standard II. Two out of seven localities are in rock massifs, i.e. workability classes 4, 5 and 6 (standard 1) and classes II and III (standard 2). This implies that the first standard is more sensitive (plasticity index, consistency index, and character of grain-size fractions) to the realization of earthwork in line with the real character of soil types.

The major advantage of the first assessment (based on standard 1) is the fact that higher sensitivity to the physical properties projected into workability classes permits more balanced pricing of earthwork. This means, for example, that if fine-grained soil of soft consistency falls into class 2, the developer spends less energy (fuel or electricity) to excavate and load the soils than in the case of stiff to hard consistency (workability class 3). However, when using standard 2 for assessment, two different layers of different consistency will be classified into workability class I and thus costs should stay the same. More intense work needed will not be projected into the budget. This way, investors and contractors do not have equal positions in pricing.

On the other hand, a simpler assessment based on standard 2 allows for fewer mistakes during engineering-geological investigations. When an engineering geologist (geotechnician) does not determine the soil consistency correctly, the mistake will show in wrong workability classification and thus the price of the planned earthwork. This simplification makes the planning faster, but does not fully consider the real physical conditions of the geological environment.

As for soil massifs, there is a bigger difference in workability classes between the two standards than it is with rocks. Workability class III (standard 2) is more compatible with classification into workability classes 4, 5 and 6 (in line with standard 1). From the point of view of semi-rocks and hard rocks and the real physical state of the geological environment, standard 2 appears as more objective than standard 1 because it considers the relationship of compressive strength of rocks.

We found that 76.7% of all layers (33 layers out of 43) were classified into the newly proposed category of an easy degree of earthwork realization difficulty that was the cheapest and technically least demanding. This is because in the studied localities there is a high occurrence of Quaternary unconsolidated sediments in the surface geological structure. Such geological structure is a general phenomenon, as sewer systems are constructed near the ground surface where more recent, unconsolidated geological structure is found. In general, it holds that the geological structure related to the occurrence of soil massifs is connected with lower workability classes and thus an easy degree of earthwork realization difficulty.

Next, in the category of a medium degree of earthwork realization difficulty there are 18.6% out of all the layers (8 out of 43), and in the most costly and most demanding category (a difficult degree of earthwork realization) there are 4.7% out of all layers (2 out of 43). Only two localities from all the case studies were situated in semi-rock and rock environments. When regional geology is related to a higher occurrence of rocks, the likelihood of this category will be higher.

When considering localities 5 and 6, we may claim that if there is a geological structure of rocks in a weathering mantle, the lower layers will contain hard rocks characteristic of higher workability classes and thus the category of a difficult degree of earthwork realization. If the weathering mantle (eluvium) is made up by semi-rocks, it is characterized by the category of a medium degree of earthwork realization. However, if the upper weathering mantle is weathered all the way to the form of loams, the environment is characteristic of low workability classes and thus the category of an easy degree of earthwork realization.

## Methods

**Figure 8:**
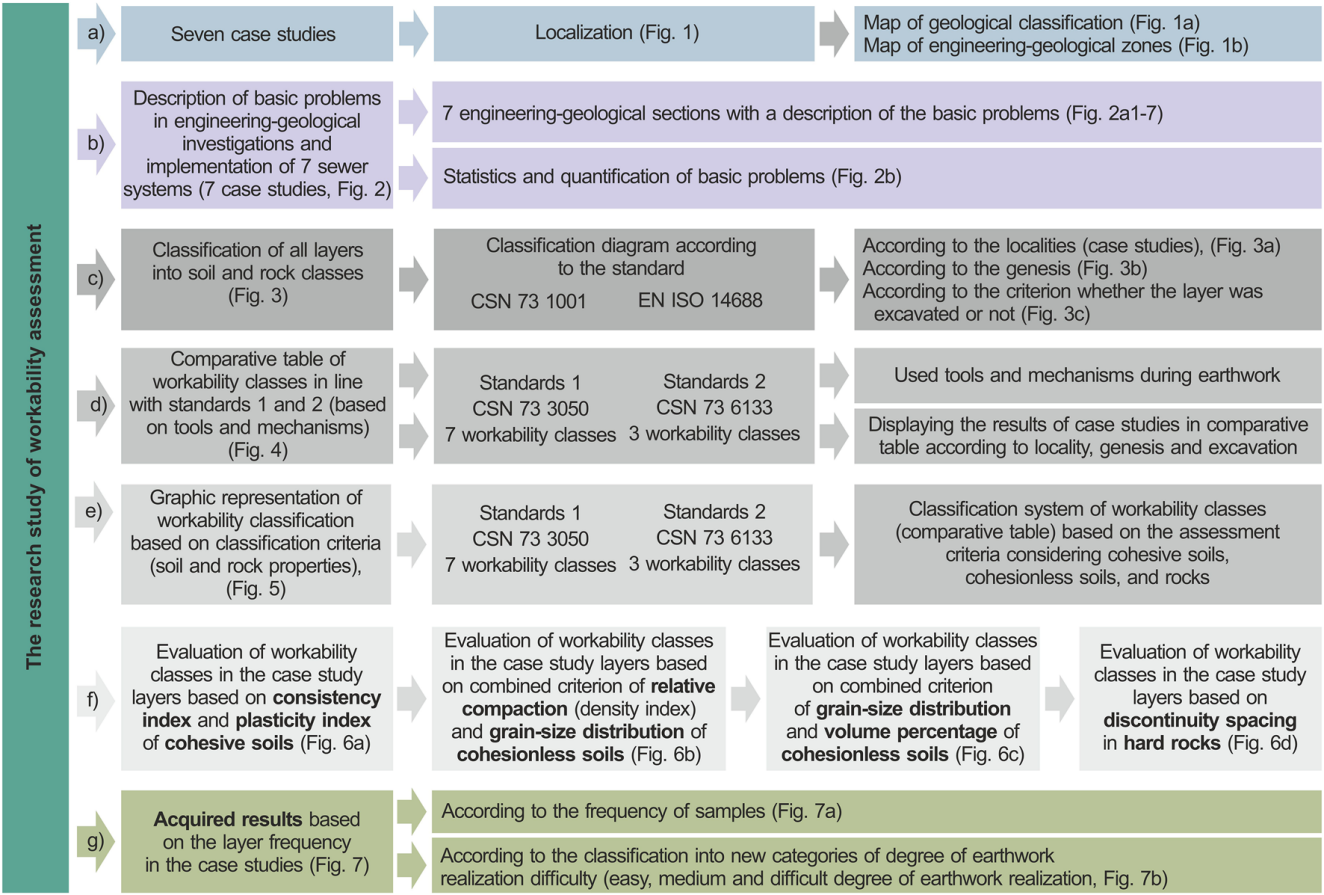
Simplified scheme of research goals and article structure; (**a**) seven case studies, (**b**) description of Statistics and quantification of basic problems in engineering-geological investigations and implementation of the seven sewer systems, (**c**) classification of all layers into soil and rock classes, (**d**) comparative table of workability classes in line with standards 1 and 2 (based on tools and mechanisms), (**e**) graphic representation of workability classes, (**f**) workability class assessment of the layers, and plasticity index (**g**) acquired results based on the layer frequency in the case studies.

The research study of workability assessment (Fig. 8) was implemented by means of 7 case studies, where each is connected to a different locality in Northern Moravia, in the north-east of Czechia (Fig. 8a). Within the evaluation, each of the seven sewer system construction projects was described for the basic engineering-geological investigation characteristics related to workability. A schematic engineering-geological section was made for each locality (Fig. 8b), describing the most relevant problems. The results were used for a statistical evaluation and quantification (Fig. 8b) of specific types of problems in engineering-geological investigations and sewer system construction.

Next, soil at all the seven studied localities was sampled and layers were classified into soil and rock type classes (Fig. 8c), which are depicted under two classification systems in line with two standards (CSN 73 1001 and EN ISO 14,688^28,29^). Each type of soil was described in all layers and related to their locality and genesis, and it was recorded whether the layer had been excavated or made part of the sewer system subsoil during the engineering-geological investigation.

Two standards were used to assess workability and to classify the soil and rock samples into the different workability classes. The first standard (CSN 73 3050^26^) classifies soils and rocks into seven classes, and the second (CSN 73 6133^27^) has only three classes. Each of those standards has its advantages and disadvantages, which will be shown based on the comparison of the seven different localities where sewer systems were constructed. A comparative table of workability for both the standards (standard 1—CSN 73 3050 and standard 2—CSN 73 6133^26,27^) was prepared, based on the tools and mechanisms used for earthwork (Fig. 8d). The workability tools and mechanisms are very important for soil and rock classification into workability classes. This conversion table enables us to compare and understand the approaches to both classifications under the different standards. The comparison was carried out for each layer of each case study locality based on workability tool and mechanisms.

Next, the classification system of workability classes was graphically represented (comparative table), as described in standards 1 and 2 based on the assessment criteria considering cohesive soils, cohesionless soils, and rocks (Fig. 8e). Discrete groups of workability classes were established in line with the graphic representation of the classification system (Fig. 8f) and based on the assessment criteria (graphs) depending on which soil or rock group was concerned (cohesive soils, cohesionless soils, or rocks). The first standard was described by means of graphs; first, workability classes of cohesive soil layers were evaluated according to the combined criteria of plasticity index and consistency index. Next, cohesionless soil layers of all case studies were evaluated according to the combined criteria of relative compaction and grain-size ratio. The evaluation that followed concerned the layers of cohesionless soils according to the combined criteria of grain-size distribution and the volume percentage, and the evaluation of rock layers based on discontinuity spacing. A similar process was adopted for the second standard. As the assessment according to the second standard was easier, it was described solely by means of text.

Finally, the frequency of all layers was evaluated (Fig. 8g) based on the newly proposed categories of earthwork realization difficulty. We also evaluated, described and explained the statistics behind the categories arising from all the layers recorded in the seven sewer system construction projects (case studies).

### Ethical approval

This article does not contain any studies with human participants or animals performed by any of the authors.

## Data Availability

The data that support the findings of this study are available from the corresponding author upon reasonable request.
